# Ethnopharmacological survey of Samburu district, Kenya

**DOI:** 10.1186/1746-4269-4-14

**Published:** 2008-05-23

**Authors:** Mark O Nanyingi, James M Mbaria, Adamson L Lanyasunya, Cyrus G Wagate, Kipsengeret B Koros, Humphrey F Kaburia, Rahab W Munenge, William O Ogara

**Affiliations:** 1 Department of Public health Pharmacology and Toxicology, University of Nairobi, P.O BOX 29053-00625 Nairobi, Kenya; 2 Samburu Integrated Resource Aid Network (SIRAN), P.O BOX 26 Maralal, Kenya; 3 Center for Public Health Research, Kenya Medical Research Institute, P.O. Box 54840, 00200 Nairobi, Kenya

## Abstract

**Background:**

Ethnobotanical pharmacopoeia is confidently used in disease intervention and there is need for documentation and preservation of traditional medical knowledge to bolster the discovery of novel drugs. The objective of the present study was to document the indigenous medicinal plant utilization, management and their extinction threats in Samburu District, Kenya.

**Methods:**

Field research was conducted in six divisions of Samburu District in Kenya. We randomly sampled 100 consented interviewees stratified by age, gender, occupation and level of education. We collected plant use data through semi-structured questionnaires; transect walks, oral interviews and focus groups discussions. Voucher specimens of all cited botanic species were collected and deposited at University of Nairobi's botany herbarium.

**Results:**

Data on plant use from the informants yielded 990 citations on 56 medicinal plant species, which are used to treat 54 different animal and human diseases including; malaria, digestive disorders, respiratory syndromes and ectoparasites.

**Conclusion:**

The ethnomedicinal use of plant species was documented in the study area for treatment of both human and veterinary diseases. The local population has high ethnobotanical knowledge and has adopted sound management conservation practices. The major threatening factors reported were anthropogenic and natural. Ethnomedical documentation and sustainable plant utilization can support drug discovery efforts in developing countries.

## Background

The Samburu pastoralists of Kenya are still among the traditional communities of the country that have retained most of their knowledge about the use of a large part of the plants in their environment for a wide variety of purposes. This knowledge is however dwindling rapidly due to changes towards a more western lifestyle, overgrazing and overexploitation of plant resources have already led to a decline of the plant material available [1].

Ethnopharmacology and natural product drug discovery remains a significant hope in the improving the poor livelihoods of rural communities. Many modern pharmaceuticals have their origin in ethnomedicine and ethnoveterinary medicine, which relies upon a local pharmacopoeia [2]. The ethnopharmacology knowledge is a holistic system approach that can serve as an innovative and powerful discovery engines for newer, safer and affordable medicines [3].

High throughput screening in industries and the isolation of many have proven to be of poor cost-effectiveness due to lack of comprehensive biological and clinical evaluation [4].

Natural products from botanical sources used in traditional medicine may combat multidrug-resistant (MDR) infectious diseases through the elucidation and validation of biological compounds with novel mechanisms of action[5].

Ethnobotanical and ethnopharmacological studies normally involve field explorations of indigenous medical knowledge and biodiversity [6].

The cultural importance of traditional medicine and physical isolation of communities both in general and from primary health cares (PHCs), are the factors that influence the dramatic use of use herbal medicines in developing countries[7,8].

Cultural acceptability of traditional practices, along with perceptions of affordability, safety and efficacy play a role in stimulating scientific research and validation of traditional medicines [9].

Ethnoveterinary medicine (EVM) include use of medicinal plants, surgical techniques and management practices [10] which forms a basis of veterinary diseases management in Samburu District. Herbal medicines are cheap and readily available in the pastoral areas but lack of sufficient scientific data on efficacy, therapeutic index, toxic effects and other pharmacological and toxicological properties to support their use [11].

Despite the fact that EVM has been very crucial for the animal healthcares of most developing countries, it has not yet been well documented and much effort is needed in research and integration activities in these countries [12].

Interdisciplinary studies to effectively combine ethnography, medical anthropology and ethnopharmacology to formulate meaningful conclusions regarding how local healers effect cure should be encouraged [13].

There are several ethnomedicinal and ethnoveterinary studies which are being carried out realizing the benefit of traditional medication to promote the cheap and safe disease management. The outcomes of these researches have immense contribution to attitude change and adaptation, though there are very few in light of Kenya's biodiversity.

The population in the District depend on livestock products for their food source and the natural vegetation as source of fuel, medicine, construction materials and other cultural needs. The overdependence on natural vegetation as food, fuel, building and medicine in Samburu District might be the cause of the cause for the environmental. Therefore, there is a need to carry out more research pertaining to documentation of useful medicinal plants in this area before they disappear, especially those which are already endangered by natural and anthropogenic activities [14-16].

In the current study we investigated and documented the local use of medicinal plants, management and extinction threats. We also compared the use of medicinal plants in treatment of human and animal diseases.

## Materials and methods

### Study area and ethnographic background

**Figure 1:**
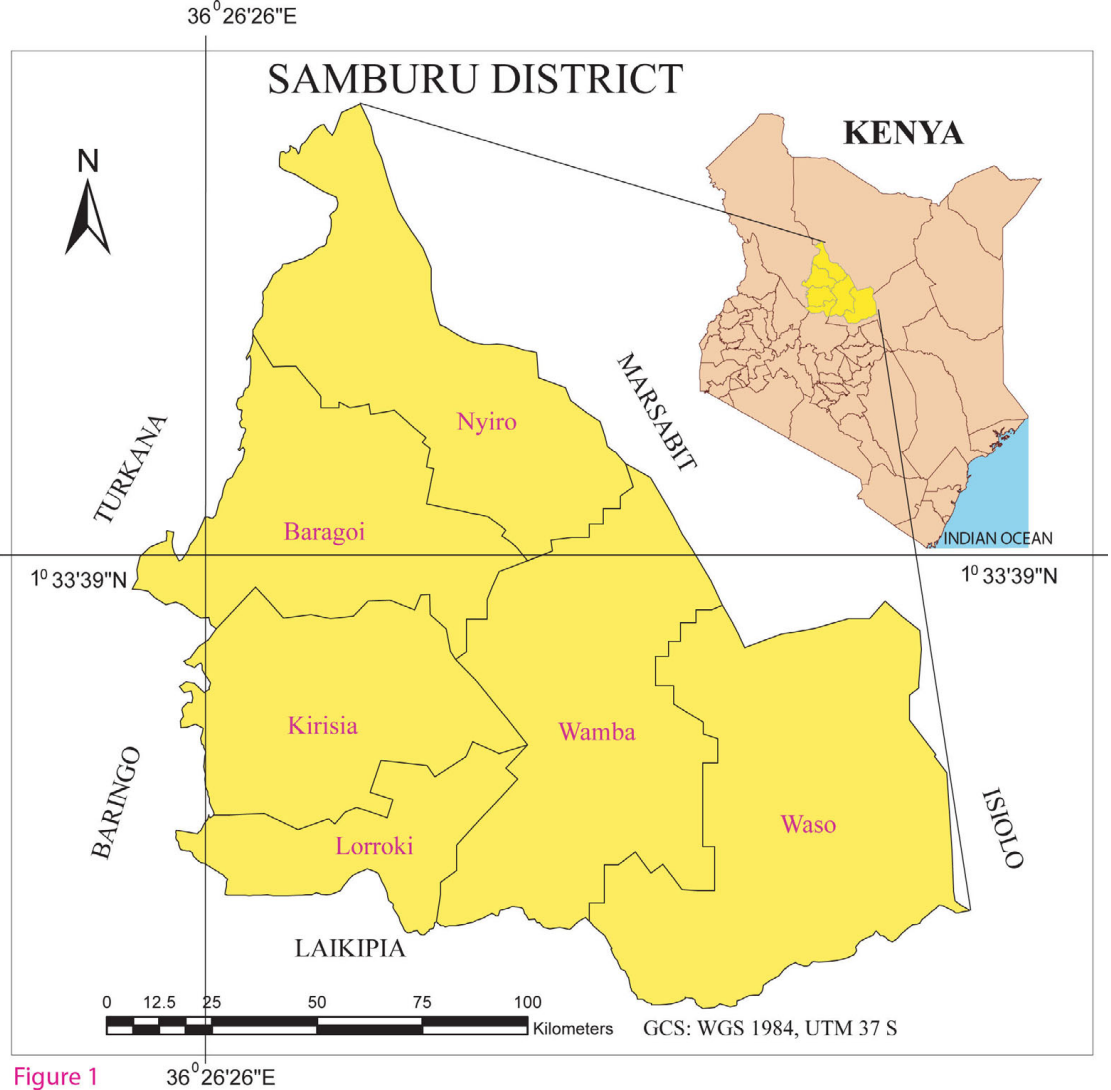
**Map of research area**. Right: Map of Kenya illustrating the geographical position of Samburu District. Left: Samburu District indicating the divisional administrative boundaries.

Samburu District is situated in the northern half of the Rift Valley Province in Kenya. It is bordered by five other Districts; Turkana (Northwest), Baringo (Southwest), Marsabit (Northeast), Isiolo (East) and Laikipia (South) respectively. It lies between Latitudes 0°40" north and 2°50" north of the equator and Longitudes 36°'20" east and 38° 10" east of the Prime Meridian (figure 1). It covers approximately 21126.5 square kilometres. It is divided into six divisions, 39 locations and 108 sub locations. It is characterised by high level plateaus, hills and the Rift valley with an altitude up to 2000 m a.s.l.

The study area has a bimodal rainfall distribution from April to May (long rains) and July to September (short rains). The dry season then extends from January to March. The mean annual rainfall is 500 mm. it has mean annual temperatures of 29°C. Samburus are the indigenous dominant ethnic group with Turkana and Maasai having settled in the area. Pastoralism is the major economic activity of the local people. The District has a population of approximately 156125 people[17]. These rural communities are almost totally dependent on forests and savannah as traditional/herbal medicine for their own health and livestock care.

### Methods

A reconnaissance survey was made from December 2006 to January 2007 to obtain an impression on vegetation characteristics of the study area. The fieldwork was done in January, February, May and August 2007.

**Figure 2:**
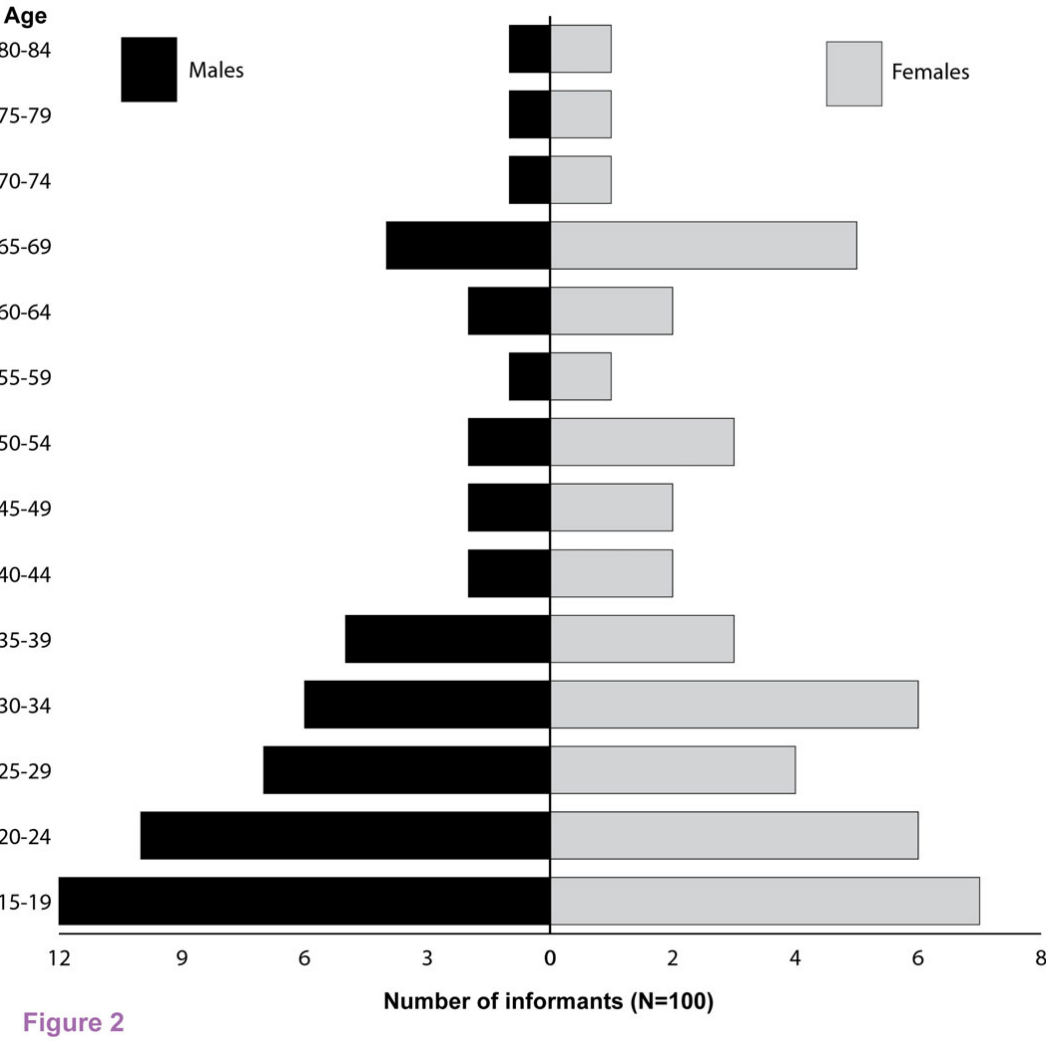
**Number of male and female informants grouped according to age category in the sample from Kirisia division in Samburu District**. Aggregated data from January-February 2007(dry season) and May-August 2007(rainy season) (N = 100 Informants).

A total of 100 informants in figure 2 were selected purposively [18] based on knowledge, attitudes and practices (KAP) survey with the help of local administrators. They included 14 traditional medicine practitioners (4 females and 10 males), 86 locals (Male: Female = 2:1). Information on knowledge depth of respondents was collected from local elderly people, opinion leaders and the local administrators. Similar responses obtained from the three groups were used to identify knowledgeable traditional healers. The respondents and traditional healers identified were consented to share their knowledge only for the purpose of this study [19].

The methods used for ethnobotanical data collection were semi structured interviews, field observation, preference ranking and direct-matrix ranking according to [18,20]. These interviews were conducted in vernacular (*Samburu*) translated by local field assistants, three different field trips were conducted.

Table 2 indicates relevant data collected on: age, sex, and occupation of informants as well as animal and human health indications treated, vernacular plant names, growth form, plant part used, methods of preparation, dosages, route of administration and possible contraindications. Threats to medicinal plants, conservation efforts, beliefs and indigenous knowledge transfer were also documented. These interviews were done in the field in order to avoid the probable confusions with regard to the identity of the medicinal plants [12]. The morphological characteristics, habitats and habits of medicinal plants were observed, photographed and recorded during and after the interviews. The key informants for purposes of ranking these species were selected randomly from among all informants [21].

**Table 2:** Data acquisition questionnaire for utilization and conservation of medicinal plants in Samburu district, Kenya

**QUESTIONNAIRE**
**PART 1: RESPONDENTS DETAILS**
Name...............................................................Sex....M/F Age...........Years.
Occupation........................................................ Level of education........................................
Location/Residence...............................................................................................................................

**Efficacy/Toxicity Data**
Type of Plant (Local name)..........................................................................................
Preparation method(s)..........................................................................................
Administration form (s)..........................................................................................
Part(s) of plant used..........................................................................................
Used on : Humans................................... Animals/Species.........................................
Route(s) of application..........................................................................................
Approximate dosage..........................................................................................
Response of Patient Good.........................Fair........................ Poor.........................
Duration of response Seconds......................... Minutes......................... Hours.........................
Complications

**PART 2: RESPONDENTS CONSENT AGREEMENT**
I.......................................................................................Hereby agree to participate in this study with my full consent and conscious and declare that to the best of my Knowledge the information that I have provided is true, accurate and complete.
Signature/Thumb print...........................................Date............./May/2007

**PART 3: RESEARCHER'S DECLARATION**
1. The following research will be undertaken with respect to the indigenous knowledge and intellectual proprietary of the Samburu Community.
2. We will at no given time initiate or conduct practices that are deemed to obtain information from the respondents by intimidation, coercion or false pretence.
3. The respondents will be informed of the intended project elaborately prior to questionnaire administration and in confidential to eliminate any degree of conspiracy.
4. We will be no under any obligation to edit or tamper the information provided by the respondents.
5. Translation and transcription will be necessary for clarification due to the language barrier.
6. The information collected will be used for the described research purpose and not any undisclosed any undisclosed intentions.

Signatory Researchers:
**1. Dr. Nanyingi M.O**..................**2. Dr. Ogara O.W**..................**3. Dr. Mbaria J.M**..................

Geographical Positioning Systems readings were also taken at the sites where each medicinal plant was collected (GARMIN, Olathe, USA). Some of the plants were identified in the field by herbalists while most were identified at the Herbarium of University of Nairobi, Botany department using specific taxonomic keys and floras[22,23].

### Data analyses

Ethnobotanical data were entered in to Excel spreadsheet and summarized using descriptive statistics [24].

Wilcoxon's test was used to determine if there was a difference age of respondents and knowledge of medicinal plants used. Chi-square test was used to evaluate the average number of medicinal plant species reported and used by each informant, to determine if there is any significant difference between female and male practitioners with respect to the knowledge and use of medicinal plants. The Spearman rank correlation test was used to determine whether there was a significant correlation between the disease reported and the number of ethnoveterinary medicinal plant species used by each informant for management of the disease. STATA 9.2 IE (Stata Corporation, College Station, Texas, USA) software was used.

## Results and discussion

### Medicinal plants diversity and Ethnobotanical knowledge

There was a highly significant difference between age of respondents and knowledge of medicinal plants (Wilcoxon's test, *p *< 0.001). The average number of medicinal plants known and used by female and male practitioners was similar (χ^2 ^= 8.262, d.f. = 13, *p *= 0.932). It was observed that informants between 58 and 77 years old mentioned more species than younger informants: 58–67 years old: about 10 per informant; 48–57: about 6; 38–47: about 5; and 28–37: 2 quoted plants, due to larger experience of older individuals. These results also agree with other previous studies[25]. It was observed that some plants had more than one vernacular name due to use of the Maasai and Turkana dialects in the area.

### Diseases treated in the study area

**Figure 3:**
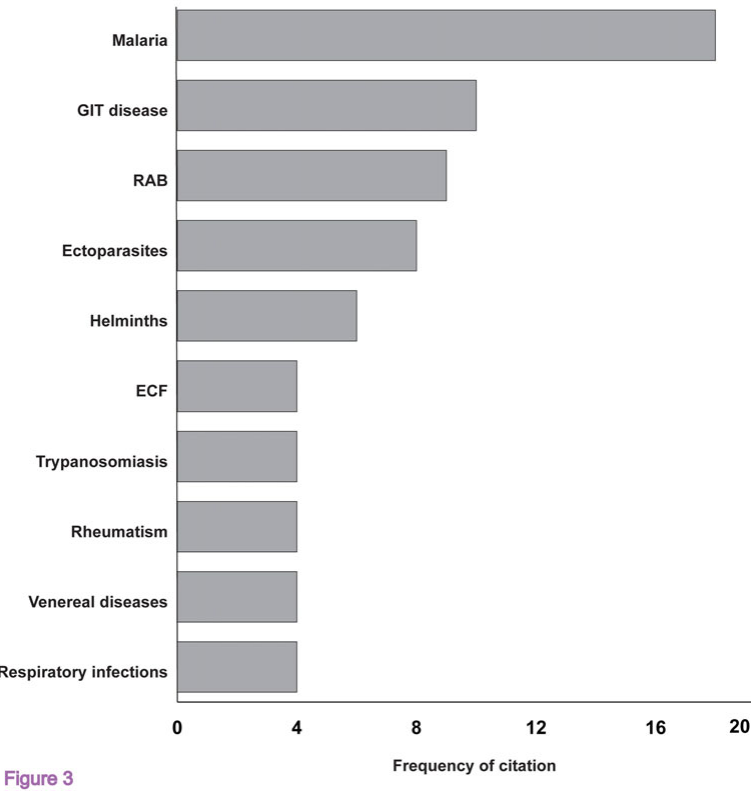
Frequency citation (n = 990) of therapeutic indications of plant remedies based on informants knowledge (n = 100) and traditional healers (n = 14).

A total of 28 animal and 26 human ailments were reported by the informants respectively. The frequency of the most cited ailments and the number of medicinal plant species used are also given in figure 3. The most frequently cited animal health problems were; Retained afterbirth (9), Ectoparasites (8), gastrointestinal disorders (5), Theileriosis (4) and helminthosis (3). Human ailments treated cited frequently included; Malaria (18), gastrointestinal disorders (10), helminthosis (5) and rheumatism (4). Respondents had good knowledge and remote diagnosis of the disease and could readily distinguish them on the basis of accepted signs and symptoms. Ailments such as convulsions, hypertension, asthma, yellow fever and infertility were beyond the scope of the present study, it was considered important to record plants that were frequently mentioned for the treatment of such health conditions (see table 1).

**Table 1:** Plants of veterinary and medical utility in Samburu District.

**Family**	**Species**	**Local name**	**Voucher no.**	**Habit**	**Part used**	**Preparation**	**Therapeutic indications**	**Route**
Anacardiaceae	*Acokanthera schimperi *Benth. & Hook.	Lmorijoi	MN 18	TR	Leaves	Hot decoction	Ectoparasites(ticks, fleas, mite)	pc
	*Adenium obesum *(Forssk.)	Lperantai	MN 40	SH	Stem bark	Powdering, Cold decoction	Ectoparasites	pc
	*Carissa edulis *(Forssk.) Vahl	Lamuriai	MN 6	SH	Roots, Leaves,	Chewing, hot/cold decoction	Theileriosis, helminthosis, rheumatism, Malaria TB, Venereal diseases(VD), Salmonellosis, Heart water	po
	*Nerium oleander *L		MN 33	SH	Leaves, Seeds	Hot decoction	URTI and GIT complications	po
	*Rhus natalensis *Bernh. ex Kraus	Lmisigiyoi	MN 8	H	Roots, leaves	Hot decoction	Malaria, fevers, TB	po
Asclepiadaceae	*Pentarrhinum inspidum *E. Mey.	Lkisuchie	MN 5	L	Leaves	Hot decoction	Anaplasmosis	po
Asphodelaceae	*Aloe secundiflora *Engl.	Sukuroi	MN 28	H	Stem	Burning and squeezing to drip hot exudate	Ectoparasites	pc
Asteraceae	*Gutenbergia cordifolia *Benth.	Lodwaporo	MN 52	SH	Leaves, Roots	powdering, hot decoction	Ticks, Giardisis	Po, pc
	*Psiadia punctulata *(DC.) Oliv. & Hiern	Labaai	MN 19	H	Leaves	Fumigation, smoke/steam bath	Ectoparasites	pc
Balanitaceae	*Balanites rotundifolia *(Tiegh.)	Sorai, ebei	MN 36	SH	Leaves	Hot decoction	GIT complications (Emetic), Eye infection	po
Boraginaceae	*Cordia sinensis *Lam	Lkweite	MN 49	SH	Flowers	Grinding, hot decoction	Malaria and fevers, Eye infection	po
	*Ehretia buxifolia*. Willd	Lkinyl	MN 2	SH	Root bark	Pounding, hot decoction	GIT complications, URTI, Malaria	po
Caesalpiniaceae	*Senna singueana *Del	Senetoi	MN 21	SH	Leaves	Grinding, hot decoction	Malaria, complicated fevers	po
Canellaceae	*Warburgia ugandensis *Sprague	Sokorioi	MN 37	TR	Stem bark, leaves	Hot decoction	helminthosis, Heart water, Ectoparasites black quarter, emetic, Trypanosomosis, ECF	po
Capparidaceae	*Capparis spinosa*.L	Lkaridangai	MN 15	SH	Root bark	Hot decoction	URTI	po
Celastraceae	*Maytenus senegalensis *(Lam.)	Laimurunyai	MN 47	SH	Leaves, roots	Hot decoction	Malaria	po
Combretaceae	*Terminalia brownii *Fries	Lbukoi	MN 32	TR	Stem bark	Hot decoction	yellow fever, GIT Complications(emetic), Trypanosomosis	po
Ebenaceae	*Euclea divinorum *Hiern	Lchingei	MN 11	SH	Seeds, Roots	Hot decoction	Malaria, Fevers, Anaplasmosis, VD	po
Euphorbiaceae	*Croton megalocarpus *Hutch.	Lmargwet	MN 26	TR	Root bark	Homogenization and decoction	Malaria, Fevers, diarrhea, Anaplasmosis, wounds.	po
	*Euphorbia candelabrum*	Mpopong'i	MN 44	TR	Leaves, barks	Stem cutting to drip Sap mix with rumen contents	URTI and GIT complications, wounds, coenurosis.	pc
	*Euphorbia herechroma *Pax	lpara	MN 53	SH	Stem sap	Stem cutting to drip Sap	Tick infestation, Ectoparasites	pc
	*Ricinus communis *L.	oldula	MN 46	SH	Leaves	Hot decoction	Malaria, fevers, RAB	
Lamiaceae	*Ajuga remota*.Benth	Lmenang'i	MN 3	H	leaves, roots	Crushing, hot decoction with soup	RAB, GIT complications, Anaplasmosis, Mastitis	po
Loranthaceae	*Odontella fischeri*	Larrudenyai	MN 43	TR	Stem bark	Hot decoction	Retained afterbirth, Wounds	po
Meliaceae	*Azadirachta indica *A. Juss.	Mwarubaini	MN 29	TR	Leaves, Stem barks	Stem cutting, pounding, Hot/cold decoction	Malaria, Fevers, GIT complications	po
Mimosaceae	*Acacia abyssinica *Hochst.	Lngingiletome	MN 45	TR	Root bark	Homogenization	Gastrointestinal distress, Lumbago and arthritis	po
	*Acacia drepanolobium*	Rangau	MN 51	TR	Root bark	Powdering, Cold decoction	RAB, Babesiosis, GIT complications	
	*Acacia nilotica *(L.) Willd.	Eluai	MN 16	TR	Root bark	Hot decoction	GIT complications, Babesiosis	po
	*Albizia anthelmintica *Brongn.	Lmungutan	MN 27	TR	Root bark, roots	Pounding, Cold decoction	Antihelmintic (Lungworms), Malaria, wounds	po
Myrsinaceae	*Myrsine africana *L.	Seketet	MN 7	SH	Seeds	Grinding, hot decoction, chewing	Helminthosis, Malaria, Wounds, TB, GIT complications	po
	*Rapanea melanophloeios*.L	Sitoni	MN 48	SH	Seeds	Grinding, hot decoction	Antihelmintic (Roundworms).	po
Myrtaceae	*Syzygium cordatum *Hochst	Loiragi	MN 39	SH	Leaves	Hot decoction	GIT complications	po
Olacaceae	*Ximenia caffra *Sond.	Ledat	MN 13	SH	Roots, leaves	Hot decoction	Malaria, Fevers, Acute URTI, Dermatitis, ulcers,	po
Oleaceae	*Olea africana *Miller	Lgeriyoi	MN 23	SH	Stem bark	Pounding, hot decoction	Helmithosis, Asthma, Rheumatism, Lumbago,	po
	*Schrebera alata*	Lkauwawa	MN 35	SH	Root bark	Pounding, chewing	Candidiasis, Toothache	po
Poacea	*Enteropogon macrostachyus *(Hochst.)	Lkujita-ongo	MN 14	L	whole plant	Hot decoction	Tryapanosomosis	po
Podocarpaceae	*Podocarpus falcatus *(Thunb.)	Masanduku	MN 38	SH	Leaves	Hot decoction, Fumigation	Measles	pc
Rhamnaceae	*Cissus quadrangularis*.L	Sukurtuti	MN 50	SH	Leaves, Fruits	Crushing, homogenizing for hot/cold decoction	Wounds, gastric ulcers, schistosomiasis, neurosis, ECF, rheumatism, epilepsy, TB, Asthma, collibacillosis	po
	*Helinus integrifolius *(Lam.) Kuntze	Lmekori	MN 12	SH	Root bark	Grinding. hot decoction, mix with milk	Arthritis, paralysis	po
	*Rhamnus stado *L	Lkukulai	MN 10	SH	leaves, fruits	hot decoction	Malaria, fevers	po
	*Scutia myrtina *(Burm. F.) Kuntz	Laturdiai	MN 20	SH	Leaves	Hot decoction	Retained afterbirth	po
Rubiaceae	*Rubia cordifolia *L.	Loitunenei	MN 9	L	Leaves, Roots	Hot decoction	URTI	po
Rutaceae	*Teclea simplicifolia *(Engl.)	Lgelai	MN 42	TR	Ro, Flowers	Hot decoction	Cerebral malaria, Fevers	po
	*Zanthoxylum usambarense *(Engl.)	Loisuk	MN 31	SH	Seeds	Grinding, Hot decoction	URTI, Malaria, Malignant catarrhal fever.	po
Salvadoraceae	*Salvadora persica *L	Sekotei	MN 41	SH	Roots	Grinding, hot decoction	RAB, ulcers, seizures, toothbrush, mange, Trypanosomosis, Brucellosis, and Anthrax.	po
Simaroubaceae	*Harrisonia abyssinica *Oliv.	Lasaramai	MN 24	SH	Roots, Leaves	Grinding, hot decoction	Abscess, ECF, Malaria, Lumbago, Rheumatism, RAB	po
Solanaceae	*Nicotiana tabacum *L.	Lkumbao	MN 34	SH	Leaves	Crushing, smoke bath, chewing	Snuff, Ectoparasites, wounds, Babesiosis, gastro-enteritis, chronic cough, gingivitis, candidosis, glossitis	Pc,
	*Solanum incanum *L.	Ltulelei	MN 22	H	Fruit	Burn and drip sap on skin	Ectoparasites	pc
Verbanaceae	*Lippia javanica *(Bur)	Sunoni	MN 4	SH	Leaves	Fumigation, decoction	Migraines, Measles	in, pc
	*Clerodendrum myricoides *(Hochst.)	Lmakutikuti	MN 25	TR	Root	Powdering, hot decoction, chewing	GIT, Lumbago, Venereal diseases.	po
Viscaeae	*Viscum tuberculatam*	Larrudenyai	MN54	SH	Root bark	Hot decoction	RAB	po
Vitaceae	*Rhoicissus tridentata *(L.F.)	Nkilenyai	MN 1	L	Leaves	Crushing, cold homogenization	URTI, Malaria	
	*Hilderbrantia sepalosa*	Nyirman	MN 30	SH	Roots	Crushing, hot decoction	URTI and GIT complications	Po

**Habit **(H – *Herb*, L – *Liana*, SH – *Shrub*, TR – *Tree*); **Therapeutic indications **(ECF – *East Cost Fever*, GIT – *Gastrointestinal*, RAB – *Retained Afterbirth*, TB – *Tuberculosis*, URTI – *Upper Respiratory Tract Infections*, VD – *Venereal Diseases*); **Routes **(in -*Intranasal*, pc – *Per cutaneous*, po – *Per *os).

Ticks (*lntunturi*) were the main cattle ectoparasites that the local people controlled using traditional plant extracts. The most frequently used plants for tick control were found to be: *Acokanthera schimperi *(*Lmorijoi*), *Adenium obesum *(Lperantai), *Aloe secundiflora *(Sukuroi),*Psiadia punctulata *(Labaai),*Nicotiana tabacam *(Lkumbao), *Euphorbia herechroma *(Lpara) represented in table 1.

The respondents classified all intestinal worms under one local name, *ntubui *and therefore use the same plant extracts for all helminths. The main species used for this were: seeds of *Myrsine africana *L (*Seketet*) (45%), *Albizia anthelmintica *(*Lmungutan*) (30%) and *Warburgia ugandensis *(*Sokorioi*) (18%).

### Medicinal plants used by the locals

**Figure 4:**
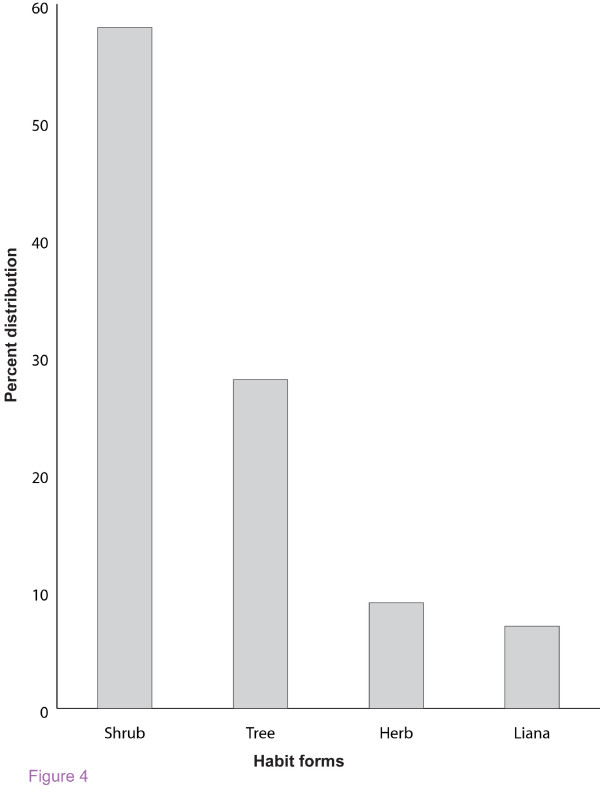
Percentage distribution of the habit growth forms of medicinal plants.

**Figure 5:**
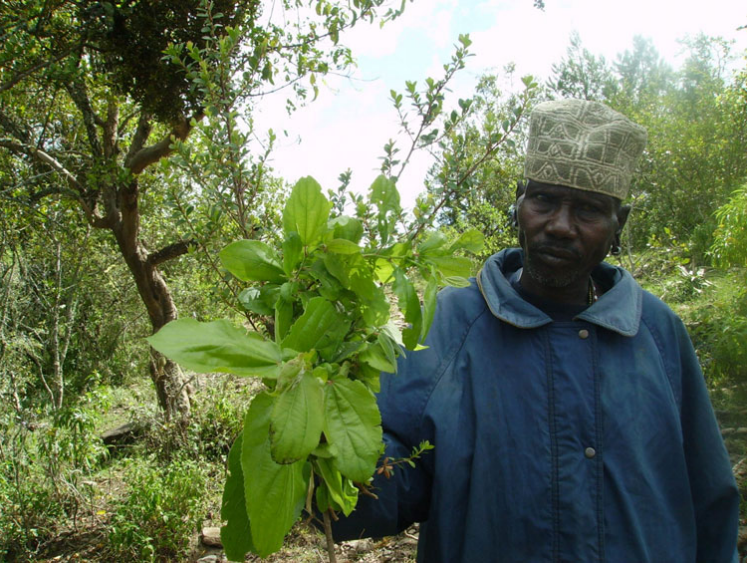
Lewaso Aplea (66 years), The most revered and knowledgeable of the remaining traditional healers in Samburu District displaying, *Ximenia caffra *Sond.(*Ledat*) and *Myrsine africana *L.(*Seketet*) during the field collection.

Fifty four (54) plant species of ethnopharmacological importance were gathered and documented throughout the study period (table 1). These medicinal plants were distributed among 50 genera and 33 families. Analysis of the growth forms of these medicinal plants revealed that, shrubs constituted the largest number or proportion with 31 species (56%), followed by trees 15 (28 %), herbs 5 (9%) and lianas 4 (7%) respectively shown in figure 4. Ethnobotanical knowledge was passed on by word of mouth. Knowledge of ailments such epilepsy, hypertension, venereal diseases, impotence, was generally restricted to the elders and traditional medicine practitioners represented in figure 5.

**Figure 6:**
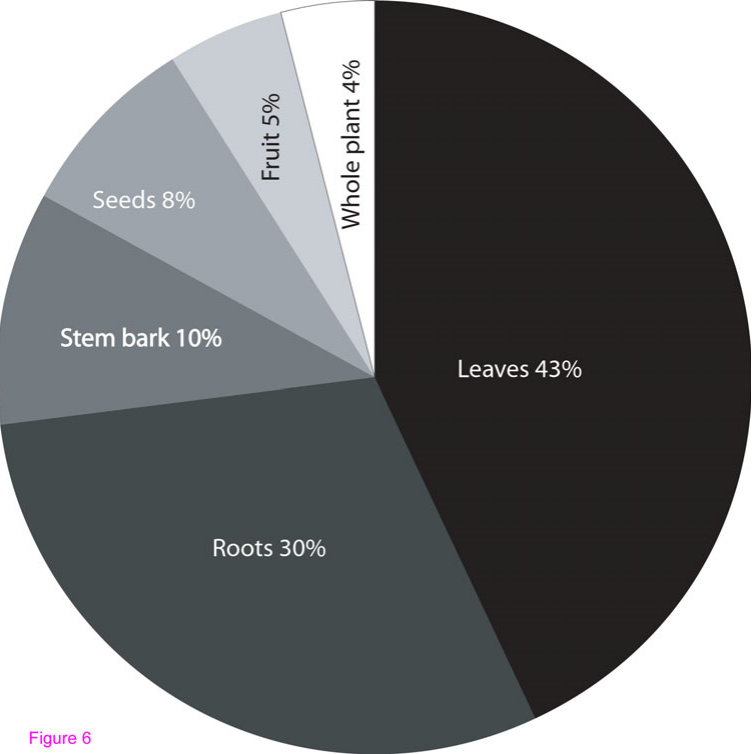
Percentage distribution of Plant parts used in Samburu District.

Leaves were the most frequently used plant parts constituting 4 %, followed by roots (3 0%), stems (10%), fruit/seeds (8%) and whole plant (4%) in figure 6. The majority of informants (42%) mentioned *Myrsine africana *L. (*Seketet*) as medicinal for the treatment of various animal and human ailments.*Seketet *was thus the most popular remedy in the study area, followed by *Carissa edulis *F. (*Lamuriai*) (5%), *Salvadora persica *L. (*Sekotei*) (30%), *Albizia anthelmintica *Brongn. (*Lmungutan*) (27%) and *Clerodendrum myricoides *Hochst. (*Lmakutikuti*) (22%).

**Figure 7:**
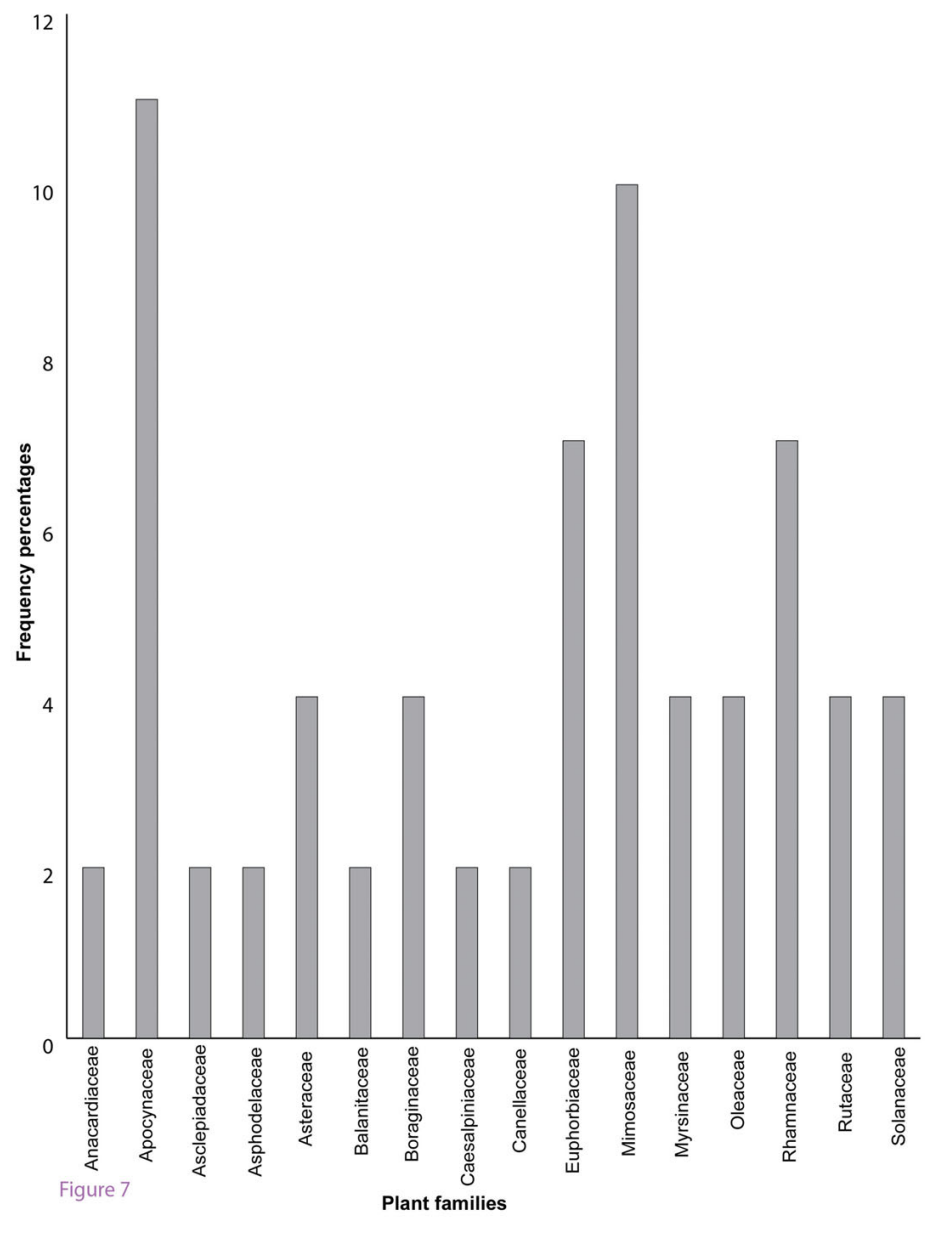
Species frequency of major plant families used in Samburu District.

The number of species frequently used in each family was cited as; Apocynaceae (6), Mimosaceae (5), Euphorbiaceae and Rhamnaceae (4) and Asteraceae (2) other families were represented by at most one species shown in figure 7.

The preparation of the medicines employed several methods; hot decoction (48%) followed by cold decoction (19.4%) and homogenization by pounding or powdering (6.5%) respectively in (table 1). The majority of these preparations were drawn from mixtures of different plant species for the treatment of a single ailment. Oral administration (8%) was the predominant route of administration followed by dermal and nasal administrations (20%).

### Medicinal plants extinction threats

Many medicinal plants in the study area were mainly threatened by anthropogenic and natural factors. The majority of medicinal plants declined due to deforestation for construction, tools, firewood, fodder, agricultural expansion and ceremonial purposes. Drought, overgrazing, bush fires had reportedly affected a significant number of medicinal plant species.

### Conservation efforts and indigenous knowledge transfer

About 47% of the informants had sufficient awareness in conserving some medicinal plant species that were relatively scarce in their surroundings. *In situ *protection of plants (fencing plants in their natural habitat, refraining from excessive cutting, debarking and uprooting and protection from fire) and *ex situ *conservation by cultivation of some plants as live fence and in nurseries were undertaken by the locals. Moreover, some of them were keen to inform responsible bodies or authorities of any illegal logging, deforestation and bush fires.

Majority of local healers preferred to collect medicinal plants solely to preserve their secrecy sometimes accompanied by the chosen family member(s). The ethnobotanical knowledge is transferred to that trustworthy family member by word of mouth rather than through a well organized written script [23]. Some of the ethnopractitioners were reluctant to pass on their plant use knowledge even to their families leading to the fragmentation and loss of the indigenous knowledge system and eventually medicinal plants [12].

This study revealed that traditional medical healers and pastoralists in Samburu District had sound knowledge of traditional medicine, from whom about 54 indications (animal and human) and a total of 56 medicinal plants of importance were recorded.

The continued reliance of Samburus' on traditional medicines is due to economic circumstances, which place modern health facilities, services and pharmaceuticals out of the reach of the majority of the population. It is also attributable to the widespread belief in the effectiveness of many traditional therapies [27].

The current investigation indicates that leaves are the most collected plant parts for medicinal purposes and this situation could be a severe threat to some rare and slowly reproducing medicinal plants. The practice of exploiting perennial plant parts, such as roots of relatively slow growing woody species, can result in a decline in both, the size and distributions of populations of the exploited species, and ultimately result in the local extinction of these populations [28].

The use of plants is evenly distributed for management of both medical and veterinary conditions this finding was contrary to earlier findings in the same geographical zone which reported insignificant veterinary use [1].

The majority of plant preparations were drawn from mixtures of different plant species for the treatment of a single ailment and similar results had reported elsewhere [26]. This was contrary to the findings of other researchers in other countries where most of the remedies were prepared from a single species [29]. This could also be ascribed to the differences in the socio cultural landscapes, indigenous knowledge on synergetic effect of different medicinal plants and vegetation types in the current study area [29].

The most frequently used methods of preparation were hot decoctions, cold decoctions, powdering and grinding respectively. The prepared medicines were mainly administered through oral (98%), dermal (1.5%), and nasal (0.5%), routes concurring with the previous findings in Ethiopia [30].

The measurements used to determine the dosages were not standardized and depended on the age and physical appearance of the patient, sociocultural explanation of the illness, diagnosis and experience of individual herbalist [27].

The naming of diseases by local people when compared to conventional systems, at times did not distinguish between diseases and symptoms of diseases. This is because local disease nomenclature is based on symptoms of diseases and not according to aetiological information [31,32].

While conducting this study, some informants raised some concern on false promises about getting the feedback. They agreed that scientific methods are better in revealing harmful effects of herbs. In the drug development research and bioprospection, biological activity based on ethnomedical uses seems as a better approach compared to randomly selected plants [33]

We are currently undertaking field trials, *in-vitro *and *in-vivo *tests of these plants for antihelmintic, antiparasitic, antiplasmodial, antibacterial and cytotoxic activities to confirm the therapeutic properties claimed by informants.

## Conclusions and recommendations

Indigenous knowledge, botanical diversity and ethnopharmacopoeia practices were recorded from Samburu District. The botanical resources were found to be under threat due to several anthropogenic and natural factors.

Disappearance of traditional medical skills was evident in the study area and this prompts for design of linguistic, anthropological and ethnographic methods in the context of ethnopharmacology to document the indigenous knowledge so as to minimize the eminent fragmentation and biodiversity loss.

The lack of standardized posology of the traditional medicines should encourage pharmacological and toxicological tests to develop formulations that can be administered in measurable dosages whose clinical efficacy can be monitored and pharmacovigilance mechanisms instituted to eliminate development of resistance to these novel compounds. Scientific feedback studies should be encouraged to instil confidence in the increasingly suspicious local populations to eliminate the apparent hostility observed among some the informants during the field research.

The data presented in this paper form a basis for further ethnopharmacological research in this region especially in studies dealing with efficacy, dosage, quality and toxicology. Those plants found empirically to be particularly effective can be used in preparation of commercial indigenous-based pharmaceuticals. We recommend that ethnopharmacologists project pharmacologic data against a backdrop of medical ethnography and anthropology. Relevant evidence generated from literature review and these biological tests will be passed back in order to improve the proper use of medicinal plants and create a good relationship for future ethnobotanical studies. The local community of Samburu District, Kenya is the owner of the traditional knowledge presented in this paper, consequently any benefits that may arise from the use this knowledge must be shared with them.

## Competing interests

The authors declare that they have no competing interests.

## Authors' contributions

MON carried out the field research, analyzed the data and wrote the manuscript, JMM and WOO designed the study, conducted fieldwork, supervised the research and revised the manuscript, CGW reviewed the manuscript and conducted the field research, KBK reviewed the manuscript and assisted in data analysis, HFK, RWM and AAL assisted in the fieldwork and taxonomic identification of the botanic specimens. All authors read and approved the final manuscript.
